# Determination of occupational safety and health factors in young workers

**Published:** 2019-08

**Authors:** Ali Nory Ghere Aghag, Parand Pourghane

**Affiliations:** ^*a*^Department of Nursing, School of Nursing, Midwifery and Paramedicine, Guilan University of Medical Sciences, Rasht, Iran.

## Abstract

**Background:**

An increase in the population in the ancient societies has enhanced the need to provide suitable employment conditions, along with the reduction of the probability of occupational accidents and diseases among young workers. In 2015, studies in 60 European countries showed that a quarter of workers were from working conditions and the risks associated with their jobs, and young workers were at greater risk than others. Most of the accidents have occurred among young workers in the retail industry due to heavy workload and high responsibilities, and also in heavy truck drivers under the age of 27. Besides the protection of workers’ safety and health, identification of vulnerable groups among them is important. Hence, this study was conducted to examine the determinants of occupational safety and health in young workers.

**Methods:**

This study covered the articles from 1994 to 2018 indexed in PubMed, Magiran, Google Scholar, SID, Scopus, Web of Science which focused on occupational safety and health of workers.

**Results:**

12528 articles addressed the issue of occupational health and safety and vulnerable among young workers. The candidate articles showed that mechanical factors such as heavy lifting, psychosocial factors such as low attention to working conditions, and workers and organizational factors such as climatic conditions in the workplace are affecting workers health and safety, as well as increased risk and job harm. The contact of workers with chemicals was associated with skin disorders, such as eczema, and lifting heavy objects caused mild back pain and high labor costs, which could cause mental harm in workers.

**Conclusions:**

According to the results, the vulnerable group among the workers with the highest number of occupational injuries and illnesses includes young people who are dropping out of education and entering the work environment, as well as trainees and novices. Therefore, in order to prevent job losses, isolation of vulnerable groups and provision of training in the work environment can be effective.

**Keywords:**

Occupational safety, Occupational health, Worker, Review research

